# Whole‐Exome Sequencing to Screen Personal Neoantigens With High Immunogenicity in Patients With Microsatellite Stability (MSS)–Advanced Colorectal Cancer

**DOI:** 10.1155/humu/3876230

**Published:** 2026-05-04

**Authors:** Dajiang Li, Wenjing Shen, Siyu Yang, Hongmei Liu, Xiao Tan, Xinrong He, Jingchao Hao, Xinqiang Yin

**Affiliations:** ^1^ School of Pharmacy, North Sichuan Medical College, Nanchong, China, nsmc.edu.cn; ^2^ School of Clinical Medicine, North Sichuan Medical College, Nanchong, China, nsmc.edu.cn; ^3^ School of Basic Medicine and Forensic Medicine, North Sichuan Medical College, Nanchong, China, nsmc.edu.cn; ^4^ School of Pharmaceutical Sciences & Yunnan Provincial Key Laboratory of Pharmacology for Natural Products, Kunming Medical University, Kunming, China, kmmc.cn; ^5^ School of Pharmacy, Kunming Medical University, Kunming, China, kmmc.cn; ^6^ School of Modern Biomedical Industry, Kunming Medical University, Kunming, China, kmmc.cn

**Keywords:** immunotherapy, MSS CRC, NRT response, personal neoantigens, whole-exome sequencing

## Abstract

To develop a personalized neoantigen therapy strategy for microsatellite stability (MSS)–advanced colorectal cancer (CRC), neoantigens from collected human CRC samples were screened, and the feasibility and effectiveness of these neoantigens in treating CRC were explored. Whole‐exome sequencing and transcriptome sequencing were performed to identify somatic mutations, RNA expression, and human leukocyte antigen alleles. Based on these data, neoantigen candidates were predicted, and their immunogenicity was evaluated. Selected neoantigens from patients elicited enhanced T‐cell responses in CRC peripheral blood lymphocytes. Mutated peptides SOX9‐V144M, ZNF169‐A275S, CDH4‐V456M, NIM1K‐T66M, and MAP3K9‐R1008Q were more effective than nonmutated ones in Patient 1. Vaccination with mutant peptides ZNF169‐A275S and CDH4‐V456M inhibited tumor growth in an autologous humanized CRC mouse model. Highly immunogenic neoantigens are strong candidates for personalized cancer therapy, showing promise for translating into effective treatments for CRC patients with advanced disease.

## 1. Introduction

Colorectal cancer (CRC) is the third most commonly diagnosed cancer and a leading cause of cancer‐related mortality worldwide [1]. Although immune checkpoint inhibitors (ICIs) have revolutionized cancer treatment, their efficacy in CRC is largely restricted to the minority of patients with microsatellite instability–high (MSI‐H) tumors, which account for less than 5% of advanced CRC cases [2, 3]. Traditional adoptive cell therapy and tumor vaccines often use nonspecific tumor‐associated antigens, which are also expressed in normal tissues, leading to suboptimal efficacy and significant side effects due to central tolerance [4, 5].

With the advent of next‐generation sequencing, patient‐specific neoantigens have emerged as ideal targets for personalized immunotherapy. Neoantigens are true tumor‐specific antigens presented only by cancer cells, enabling the immune system to distinguish malignant from healthy tissue and minimizing adverse events [6]. The likelihood of neoantigen formation correlates with mutation frequency [7]. CRC exhibits an average mutation density of ~10 mutations/Mb [8], and effective neoantigen‐specific antitumor immune responses have been observed in MSI‐H CRC, which is associated with high tumor mutation burden [9]. However, most patients with microsatellite stability (MSS) CRC do not benefit from ICIs [10]. Thus, there is an urgent need to develop methods for identifying neoantigens in MSS CRC and facilitating personalized treatment options for this subtype.

In the present study, four patients with MSS CRC were recruited to evaluate the potential of neoantigen‐based immunotherapy. Tumor tissue samples and peripheral blood mononuclear cells (PBMCs) were collected for neoantigen identification, and their immunogenicity and antitumor effects were assessed. Our findings suggest that developing personalized immunotherapies based on neoantigens may be feasible in MSS CRC.

## 2. Materials and Methods

### 2.1. Tumor Tissue Collection and PBMC Preparation

The patients were included based on the following inclusion criteria: (1) diagnosed with CRC, with TNM stages of IIIb–IV; (2) male or female aged between 18 and 75 years old, with at least one radiographically measurable lesion; and (3) patients who had not received any treatment before. We collected tumor tissue samples from a total of four patients who met the above criteria from the Cancer Hospital of Yunnan Province, affiliated with Kunming Medical University. To protect patient privacy, the patients were randomly coded as Patient 1 (P1), Patient 2 (P2), Patient 3 (P3), and Patient 4 (P4). The clinical characteristics of the four patients are listed in Table 1.

**Table 1:** Clinical characteristics of the four patients.

Patient ID	Age	Sex	Cancer diagnosis	Pathology	Tumor stage	Metastatic sites	MSI
P1	54	M	Colon	Adenocarcinoma	T4N0M1 (IV)	Liver	MSS
P2	72	M	Rectum	Adenocarcinoma	T3N1M1 (IV)	Liver, lung, peritumor lymph node	MSS
P3	67	F	Colon	Adenocarcinoma	T3N2M1 (IV)	Left adnexa, tumor distal lymph nodes	MSS
P4	58	M	Colon	Adenocarcinoma	T4N2M1 (IV)	Lung, peritumor	MSS

Abbreviations: F, female; M, male; MSI, microsatellite instability; MSS, microsatellite stability.

Tumor samples from patients with CRC were obtained from biopsy specimens. One fragment of each sample was formalin‐fixed and paraffin‐embedded. The remaining tissue was snap‐frozen in isopentane and stored in vapor‐phase liquid nitrogen.

PBMCs were collected for immunological monitoring or to obtain samples. These cells were separated by density gradient centrifugation using Ficoll‐Paque solution (Amersham, Cytiva) and collected from peripheral blood samples of CRC patients, resulting in the generation of immature dendritic cells (DCs).

### 2.2. Whole‐Exome DNA and RNA Sequencing (RNA‐seq)

DNA extracted from fresh, pretreatment tumor tissue and corresponding blood samples was purified and subjected to hybridization using the Agilent SureSelect Target Enrichment System kit (Tiangen Biotech Co., Ltd.), according to the manufacturer’s instructions, and paired‐end multiplex sequencing of samples was performed on the Illumina NovaSeq 6000 sequencing platform. Fastp (v0.23.2) was used to control the quality of raw sequencing data and filter out low‐quality data. The filtered clean reads were then aligned with the GRCh37/hg19 human reference genome using the Burrows‐Wheeler Aligner (BWA‐0.7.12), generating a Sequence Alignment/Map (SAM) file. Alignment data were converted, sorted, and indexed using SAMtools (v1.3). Duplicates were then marked with SAMBLASTER (0.1.22) to reduce bias. The variations in the DNA sequencing results were removed using GATK (v.4.1.3.0).

RNA‐seq generates FASTQ format end‐of‐read results (paired‐end, 150 bp) on the Illumina NovaSeq 6000 sequencing platform. Fastp (v0.23.2) was used to control the quality of RNA‐seq data and filter out low‐quality data. The filtered clean reads were then aligned with the GRCh38/hg38 human reference genome using the STAR software (Version 2.6.0a), thereby creating a SAM file. Based on SAMtools (v1.3), the SAM file was converted into a BAM file and indexed. GATK (v.4.1.3.0) was used to detect differences in RNA‐seq results.

### 2.3. Identification and Annotation of Somatic Mutations

VarDict (Version 1.7.0) was used to detect somatic mutations, encompassing single nucleotide variants (SNVs) and insertions and deletions (indels). Subsequently, the mutations were annotated using the Ensembl Variant Effect Predictor (v97) software. The tumor mutation load (TMB) was defined as the number of all nonsynonymous mutations and indels detected per Mb of the genome.

### 2.4. Human Leukocyte Antigen (HLA) Typing

The patients’ HLA genotype was assessed using Polysolver (1.0) [11] and BWA‐HLA (1.3) based on the DNA sequencing data. If Polysolver identified the same genotype in both tumor and normal samples, the result from Polysolver was adopted as the HLA genotype. Where this was not the case, the outcome of BWA‐HLA was referenced. If BWA‐HLA identified a consistent genotype across both sample types, its result was used. When neither method identified agreement, results from both Polysolver and BWA‐HLA were considered. If both methods identified the same genotype in the normal sample, this result was used for the HLA genotype. Discrepancies between Polysolver and BWA‐HLA resulted in the adoption of Polysolver’s normal sample result as the HLA genotype, though it was marked as having low confidence. The patients’ HLA genotypes are listed in Table S1.

### 2.5. Neoantigen Identification

Using a specialized pipeline for neoantigen detection called TruNeo (YuceBio) [12], nonsynonymous somatic mutations such as SNVs, indels, and frameshifts were analyzed to predict potential neoantigens. First, mutant peptides measuring 8–11 and 12–15 amino acids were generated for major histocompatibility complex (MHC) Class I– and Class II–restricted neoantigen predictions, respectively. The binding affinity of peptides for HLAs was predicted using the NetMHCpan 3.0 and NetMHCIIpan 3.2 [13]. The expression levels of mutated genes were derived from RNA‐seq data, measured in transcripts per million. Each peptide was prioritized based on HLA binding affinity, expression level, similarity to self‐peptides, and mutation allele frequency. Peptides with a priority score of > 0 were selected as neoantigen candidates, and %rank values of < 0.5 indicated strong binding peptides, while values of > 0.5 were deemed weak binders.

### 2.6. Microsatellite Instability (MSI) Status Analysis

The MSI status of a sample was analyzed using the MSI sensor software. By extracting reads in the microsatellite (MS) bed region of the sample, the length distribution of each MS (i.e., the allelic distribution of each MS locus) was counted and compared with the internal MS locus allelic distribution data of the software. The MSS (stable type) status and MSI (unstable type) status of MS loci were judged from the germline level, and the distribution of MS in the sample was statistically analyzed. The proportion of MSI loci to total MS loci was used to determine the MS status of the sample. When the MSI score was ≥ 20, the sample was considered MSI‐positive (MSI‐H). The MSI status of the four patients is listed in Table 1.

### 2.7. Synthesis of Candidate Peptides and Preparation of Vaccines

The candidate peptides were synthesized by GL Biochem (Shanghai) Ltd. with a purity of ≥ 98% and were confirmed by mass spectrometry. The polypeptide measured 29 amino acids, centered on the mutation site, with 14 amino acids added before and after the polypeptide, respectively. The lyophilized peptides were stored as isocyanates at −80°C. The neoantigens of P1 for patient‐derived xenograft (PDX) mouse immunotherapy (ZNF169‐A275S and CDH4‐V456M) were mixed, respectively, with 0.5 mg of polyinosinic:polycytidylic acid (poly I:C) (Shanghai Yuanye Bio‐Technology Co., Ltd.).

### 2.8. Recombinant Minigene‐pcDNA3.1 Plasmids

Minigenes encoding the mutated amino acid together with upstream and downstream flanking native sequences (total length, 29 amino acids) were constructed for each mutation of interest. Multiple minigenes were arranged in tandem without additional linker sequences and synthesized (Sangon Biotech Co., Ltd., Shanghai). Each tandem minigene construct was inserted into the pcDNA3.1 vector using *EcoRI* and *BamHI* restriction sites to generate minigene‐pcDNA3.1 plasmids. The procedure was performed strictly following the manufacturer’s instructions.

### 2.9. Establishment of Stable Transfectants of SW480

SW480 cells in the logarithmic growth phase were stably transfected with minigene‐pcDNA3.1 plasmids using Lipofectamine and PLUS reagent (Invitrogen, San Diego, California, United States) according to the manufacturer’s instructions. G418 (Invitrogen, San Diego, California, United States) was used to select positively transfected cells. Transfection efficiency was confirmed by RT‐PCR.

### 2.10. Induction of Neoantigen‐Reactive T Cells (NRTs) In Vitro

PBMCs from patients with CRC were isolated, and monocyte‐derived DCs were generated as previously described. NRTs were generated with minor modifications. After 7 days of coculture with peptide‐pulsed autologous DCs, lymphocytes were restimulated with peptide‐pulsed autologous DCs in medium containing 10 ng/mL IL‐7, followed by supplementation with 50 IU/mL rhIL‐2 (Thermo Fisher) 72 h later. Lymphocytes were restimulated at 7‐day intervals in the same manner. Half of the medium was replaced every 3 days with fresh medium containing 50 IU/mL rhIL‐2. After three rounds of stimulation, T‐cell responses were assessed by interferon gamma (IFN‐*γ*) enzyme‐linked immunospot (ELISpot) and cytotoxicity assays. To further validate peptide specificity and endogenous processing, T cells primed with synthetic peptides were cocultured with SW480 cells stably expressing tandem minigenes. T‐cell activation was evaluated by IFN‐*γ* ELISpot and CCK‐8 cytotoxicity assays as described below.

### 2.11. ELISpot Assay

IFN‐*γ* in neoantigen‐specific T cells was detected using an ELISpot kit (Mabtech) [14]. DCs were cultured as previously described [15]. Stimulation of T cells isolated from PBMCs with autologous DCs pulsed with individual neoantigen peptide (10 *μ*g/mL) and IL‐7 (10 ng/mL, PeproTech, Inc.) was performed in a 24‐well plate. On Day 3, IL‐2 (5 *μ*g/mL, PeproTech) was introduced. Half of the medium was replaced, and cytokines were supplemented every 3 days, following the procedure outlined in a previous study [16]. After 10 days, the IFN‐*γ* response from the prestimulated T cells against neoantigens was assessed using ELISpot assays with a Human IFN‐*γ* ELISpot kit (Mabtech). The ELISpot plates underwent five washes with PBS. Prestimulated T cells combined with DCs, which had been pulsed with neoantigen peptides, were positioned into separate wells of the plates and left to incubate at 37°C for 18–24 h under conditions of 5% CO_2_. Following a further five washes with PBS, the plates were treated with 100 *μ*L/well of antihuman IFN‐*γ* (7‐B6‐ALP) for further incubation for 2 h at 37°C. The plates were then washed five times with PBS and incubated with BCIP/NBT‐plus substrate at room temperature. Plates were then subsequently scanned using an ELISpot CTL Reader (Cellular Technology, Inc.). The data were analyzed using ELISpot software (AID). T‐cell reactivity was considered positive for spots that were more than double the size of those in the no‐peptide control (medium only).

### 2.12. T‐Cell Receptor (TCR) Sequencing and Neoantigen‐Specific TCR Clone Analysis

Posttreated peripheral blood collected from P1 was incubated with the patient’s ELISpot‐positive peptides. Cells were harvested after a 2‐week incubation for RNA extraction. The TCR library, assembled using cDNA reverse‐transcribed from individual RNA samples, was subjected to high‐throughput sequencing. Consequently, mature clonal diversity, average clonal frequency, TCR convergence, and enriched clones were employed to analyze complementarity‐determining region 3 (CDR3) of the T‐cell receptor beta (TCR‐*β*) chain.

### 2.13. Mouse Tumor Implantation and Neoantigen Vaccination

All experimental procedures and animal protocols were approved by the Ethics Committee of North Sichuan Medical College (Nanchong, China) and were in compliance with all relevant ethical regulations (IACUC Issue No. 2022‐0038). All procedures were designed in accordance with the ethical standards of the National Research Council to minimize animal suffering and the number of animals used. The animals used in this study were obtained from the Animal Center of North Sichuan Medical College (SCXK(川)2018‐18). The mice (n = 10 per group) were maintained at a temperature of 20 ± 2°C, with a humidity level of 55% ± 10%. They were exposed to a light intensity of 350 lux (at bench level) and a 12/12‐h light/dark cycle. All mice had access to a standard rodent diet and water ad libitum. During the feeding period, the health of the animals was monitored twice daily, and no adverse events were observed.

PDX was established as previously described [17, 18]. In brief, tumor fragments (20–30 mg) of P1 were subcutaneously implanted into the right flank of 8‐week‐old male NOD scid gamma (NSG) mice using a trocar. After the outgrowth of the primary tumor, the tumors were harvested, digested in collagenase/hyaluronidase solution, and injected subcutaneously into NSG mice in Matrigel. For tumor growth experiments, 3 × 10^6^ digested PDX cells were implanted subcutaneously in 100 *μ*L of Matrigel into the right flank of NSG mice. Once tumors were established, mice received 3 × 10^6^ autologous PBMCs intravenously. After 3 days, the mice were injected subcutaneously with the vaccine (0.3 mg of ZNF169‐A275S or CDH4‐V456M mixed with 0.5 mg of poly I:C) twice a week for 3 weeks. The tumor growth rates were monitored by measuring tumor length (*L*) and width (*W*) using a digital caliper three times a week. Volume was calculated as follows: 0.50 × *W* ^2^ × *L*. The set of humane endpoints used to determine if an animal needed to be immediately euthanized prior to the scheduled experimental endpoint is listed below: physiological indicators such as significant weight loss, persistent pain, severe infection, organ failure, etc.; behavioral changes such as lack of appetite, reduced activity, abnormal posture, excessive licking or biting, etc.; experimental progress: if the experiment has reached its expected goals or cannot continue, consideration should also be given to ending the experiment; and tumor‐related symptoms: (1) ulcers and necrosis: ulcers or necrotic areas on the surface of the tumor can cause pain and infection; (2) functional impairment: if the tumor causes paralysis, difficulty in movement, or other functional impairments in the mouse, termination of the experiment should be considered; and (3) compression symptoms: compression of surrounding tissues or organs by the tumor may lead to symptoms such as breathing difficulties or urination problems. During the course of the experiment, no experimental animals exhibited the aforementioned conditions. The mice were sacrificed on Day 80 after tumor inoculation. The survival rate was calculated as the percentage of mice alive on Day 80. In the experiment, all mice were initially anesthetized with pentobarbital sodium (60 mg/kg, intraperitoneal injection), followed by euthanasia through cervical dislocation.

### 2.14. In Vitro T‐Cell Cytotoxicity Assay

Cytotoxic activity of neoantigen‐specific T cells was assessed using the CCK‐8 assay. Target cells included T2 cells pulsed with mutated peptides (20 *μ*M) and SW480 cells stably expressing minigenes. Effector cells were cocultured with target cells at the indicated ratios. The percentage of specific lysis was calculated as follows: cell cytotoxicity (*%*) = [1 − (Ae − Ab)/(Ac − Ab)] × 100, where Ae is the absorbance of the experimental group, Ac is the absorbance of the control group, and Ab is the absorbance of blank wells.

### 2.15. Ex Vivo T‐Cell Restimulation and Functional Assessment

To evaluate the specificity and effector function of vaccine‐induced T cells, splenocytes and tumor‐infiltrating lymphocytes (TILs) were harvested from mice that exhibited tumor regression. Single‐cell suspensions were prepared from spleens by mechanical dissociation and red blood cell lysis. Tumors were minced and digested in collagenase/hyaluronidase solution to obtain TILs. Cells were restimulated ex vivo for 7 days with autologous DCs pulsed with the corresponding vaccine peptide (ZNF169‐A275S or CDH4‐V456M), with the irrelevant control peptide, or with SW480 cells stably expressing the tandem minigene encoding the mutated epitope. IFN‐*γ* production was measured by ELISpot, and cytotoxic activity against peptide‐pulsed T2 cells or minigene‐transfected SW480 cells was assessed using the CCK‐8 assay as described above.

### 2.16. Statistical Analysis

In the present study, GraphPad Prism 6 software (GraphPad Software, Inc.) was used to perform the statistical analysis. The two‐tailed Student’s *t*‐test was used to confirm the significance of differences in means. Tumor sizes across groups were compared using the Mann–Whitney *U* test. Survival analysis was performed through Kaplan–Meier curves. A *p* < 0.05 was considered to indicate a statistically significant difference.

## 3. Results

### 3.1. Somatic Mutation Screening and Neoantigen Prediction

**Figure 1:**
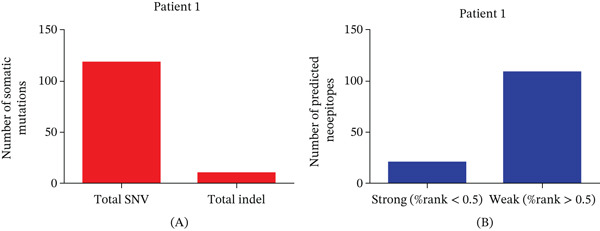
In four CRC patients, the number of somatic mutations and corresponding predicted neoantigens in P1 was examined. (A) Both whole‐exome sequencing and RNA‐seq were conducted on P1. We identified tumor‐specific nonsynonymous somatic mutations, with the count of somatic mutations in P1 indicated. (B) Neoantigens were predicted for P1. We present the number of neoantigens, along with the classification of strong binders (*%*rank < 0.5) and weak binders (0.5 < *%*rank < 2) for each individual.

Four patients diagnosed with advanced CRC were enrolled in the present study. From each participant, tumor tissue and peripheral blood cell samples were obtained to conduct whole‐exome sequencing and RNA‐seq analyses. Through these methods, an average of 142 (range, 106–175) nonsynonymous somatic mutations were identified per patient (Figure 1A, Figure S1A). These mutations were then analyzed to predict potential neoantigens that could bind to MHC Class I or II molecules. Figure 1A and Figure S1A show the total number of nonsynonymous mutations found within the exonic regions of each patient’s tumor. From these data, 93, 112, 78, and 126 MHC Class I–restricted neoantigens were predicted from P1, P2, P3, and P4, respectively. In addition, 37, 46, 28, and 49 MHC Class II–restricted neoantigens were identified from P1, P2, P3, and P4, respectively (Table S2).

Among the predicted neoantigens, an average of 19 were categorized as strong binders, exhibiting a %rank value of > 0.5, indicating a higher likelihood of binding to MHC molecules. Conversely, an average of 123 neoantigens were classified as weaker binders, showing a %rank value of > 0.5 (Figure 1B, Figure S1B). This information is crucial for understanding the potential immune response elicited by these neoantigens and may guide future immunotherapeutic strategies. From each patient, 10 long peptides (29 amino acids in length) that are potentially associated with mutations and exhibit strong HLA binding affinity were selected. These peptides were then chemically synthesized (Table 2).

**Table 2:** In silico prediction of mutations in four CRC patients with strong HLA affinity.

Patient	Gene	HLA restriction	Mutated sequence	Substitution (WT, AA#, Mut)
P1	SOX9	A∗33:03	KNKPHVKRPMNAFMMWAQAARRKLADQYP	V144M
	EVPL	DRB1∗11:01	RARVWEMLNRERTAQQAREEEARRLRERI	R1550Q
	ZNF169	C∗03:04	PYLCPECGRRFSQKSSLSIHQRKHSGEKP	A275S
	TBCD	A∗33:03	TNVRDAACYVCWAFTRAYEPQELKPFVTA	A475T
	CDH4	C∗03:04	SVRTDPVTNFGMVTMVKAVDYELNRAFML	V456M
	NIM1K	A∗33:03	TQDMSQDEKVVREIMLGKRIGFYRIRGEI	T66M
	STRN4	B∗44:02	LGSGEDGEGAPDPRWCTVDGSPHELESRR	R336W
	MTMR9	DRB1∗11:01	YNKELQAKVNILRRHLAELETEDGMQESP	Q535H
	RPS6KA2	C∗03:04	MAATYFALNRTPQALRLRPVLSSNLAQRR	P709L
	MAP3K9	DRB1∗11:01	DSTLERPKTLEFLPQPRPSANRQRLDPWW	R1008Q

P2	E2F3	A∗11:01	NLFGPFVNLLPPLLKEDYLLSLGEEEGIS	Q431K
	CTDP1	DRB1∗09:01	QQMSNKGIFHFQLGQGEPMLHTRLRPHCK	R216Q
	AMDHD2	DRB1∗09:01	VWSGATFITHLFNATLPFHHRDPGIVGLL	M237T
	SUGT1	A∗11:01	QFVADVKNLYPSSSTYTRNWDKLVGEIKE	P282T
	IMMP2L	DRB1∗11:01	IVRTIGHKNRYVKVLRGHIWVEGDHHGHS	P114L
	TENM3	B∗40:01	SPDGTLYIADLGNIQIRAVSKNKPLLNSM	R1494Q
	ADGRG7	DRB1∗11:01	KVLWKNNQNLTSTKEVSSMKKIVSTLSVA	K663E
	FBXO41	A∗11:01	YLDTRTLLHAAEVCQDWRFVARHPAVWTR	R577Q
	ARFGEF2	A∗11:01	YYNMNRIRLQWSRIRHVIGDHFNKVGCNP	W1135R
	ARHGAP10	DRB1∗11:01	SFFQGMFTFYHQGHGLAKDFNHYKMELQI	E216G

P3	ASTN1	DRB1∗15:01	GMDLTPGSDNAKLSVMNKYKDNIIATSPV	L297V
	ASPH	DRB1∗15:01	EKFPETTGCRRGQIRYSIMHPGTHVWPHT	K666R
	CENPI	A∗33:03	LGPANVRPLKRKWNFLSVIPVLNSSSYTK	S306F
	AGK	A∗33:03	TAGLCLLTWGGHWLHGKHCDNLLRRAACQ	Y30H
	CCTBL2	A∗33:03	AENSGDGTAFVVLLKEALLEQAEQLLKAG	T112K
	NAP1L3	DRB1∗15:01	PRVVPNASFFNFFSRPEIPMIGKLEPRED	P445R
	CDC16	A∗33:03	CRKLKKYAEALDYHCQALVLIPQNASTYS	R469C
	XPNPEP3	DRB1∗15:01	ETMFTSKAPVEEAFHYAKFEFECRARGAD	L282H
	QKI	DRB1∗15:01	RHDMRVHPYQRIVTTDRAATGN∗	A334T
	OPRL1	A∗33:03	KACFRKFCCASALRWDVQVSDRVRSIAKD	R341W

P4	PKHD1	B∗35:01	LKIKDKNKFYFPSLKPRKDLGKVVCPELD	Q3331K
	PLXNB2	B∗35:01	AGGTTLTIHGTHLDMGSQEDVRVTLNGVP	T922M
	WWOX	B∗35:01	LPWSLTKDGLETTFHVNHLGHFYLVQLLQ	Q230H
	CDH12	B∗35:01	TDADDPTYGNSARVIYSILQGQPYFSIDP	V199I
	OR5L2	B∗35:01	YIYCRPSSGNSGDVVKVATVFYTVVIPML	D271V
	COL19A1	B∗35:01	AKGEKGSDGPPGKPRPPGPPGIPFNERNG	G810R
	OR52K2	DRB1∗13:01	QEARYKAFGTCVSHMGAILAFYTTVVISS	I247M
	P2RX5	B∗35:01	FQDIALEGGVIGINSEWNCDLDKAASECH	I259S
	STT3A	B∗35:01	ARIFIIMYGVTSMYLSAVMVRLMLVLAPV	F399L
	IRF2BP2	B∗35:01	PPPPTASPHSNRTTLPEAAQNGQSPMAAL	P398L

### 3.2. Neoantigens Elicit Enhanced NRT Responses In Vitro From Patients With CRC

**Figure 2:**
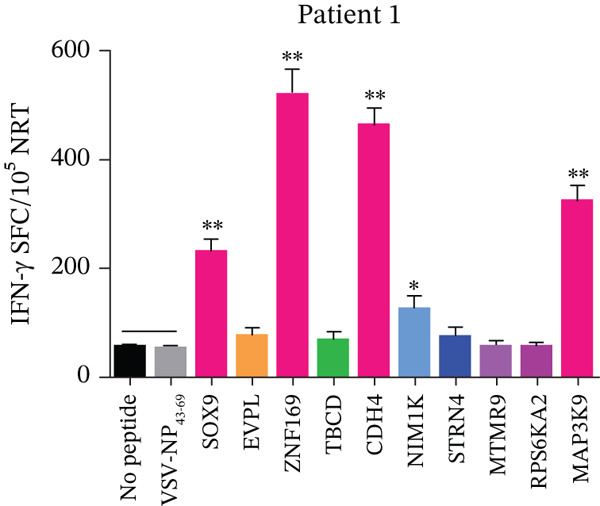
Evaluating the immunogenicity of neoantigens from patients with CRC. Autologous PBMCs were stimulated with candidate mutated peptides every 3 days in the presence of IL‐2. On Day 10, T‐cell responses to each antigen were measured by an IFN‐*γ* ELISpot assay. The PBMCs were obtained from P1 with CRC. No peptide (medium only) or VSV‐NP_43-69_ (STKVALNDLRAYVYQGIKSGNPSILHI) stimulation was used as a control. Data are presented as mean ± SD of three independent experiments.  ^∗^ *p* < 0.05 and  ^∗∗^ *p* < 0.01 compared with IFN‐*γ* production by PBMCs stimulated without peptide or with VSV‐NP_43-69_.

The PBMCs from P1, P2, P3, and P4 were used to pinpoint neoantigens exhibiting high immunogenicity. From each patient, 10 long peptides (29 amino acids in length) associated with hypothesized mutations and demonstrating strong HLA affinity were selected for chemical synthesis. The immunogenicity of these peptides in cancer patients was determined using a straightforward and swift method, which was slightly adjusted. The immunogenicity assessment of each synthetic polypeptide was conducted using the PBMCs derived from the respective patient. The ELISpot assay results indicated that compared with the medium (no peptide) or the unrelated peptide VSV‐NP_43-69_ (STKVALNDLRAYVYQGIKSGNPSILHI), the neoantigen candidates SOX9‐V144M, ZNF169‐A275S, CDH4‐V456M, NIM1K‐T66M, and MAP3K9‐R1008Q from P1 (Figure 2); CTDP1‐R216Q, SUGT1‐P282T, IMMP2L‐P114L, ADGRG7‐K663E, FBXO41‐R577Q, and ARFGEF2‐W1135R from P2; ASTN1‐L297V, CENPI‐S306F, AGK‐Y30H, CCTBL2‐T112K, CDC6‐R469C, XPNPEP3‐L282H, and OPRL1‐R341W from P3; and PKHD1‐Q3331K, PLXNB2‐T922M, CDH12‐V199I, OR52K2‐I247M, and IRF2BP2‐P398L from P4 induced peptide‐specific T‐cell responses, as shown by ELISpot assay (Figure S2A–C). In particular, SOX9‐V144M, ZNF169‐A275S, CDH4‐V456M, and MAP3K9‐R1008Q from P1; CTDP1‐R216Q, SUGT1‐P282T, and FBXO41‐R577Q from P2; ASTN1‐L297V, CENPI‐S306F, and CDC6‐R469C from P3; and PKHD1‐Q3331K and OR52K2‐I247M from P4 manifested distinct peptide‐specific T‐cell responses (Figure 2, Figure S2A–C).

### 3.3. Mutated Peptides Elicited an Enhanced NRT Response Compared With Their Wild‐Type (WT) Counterparts

**Figure 3:**
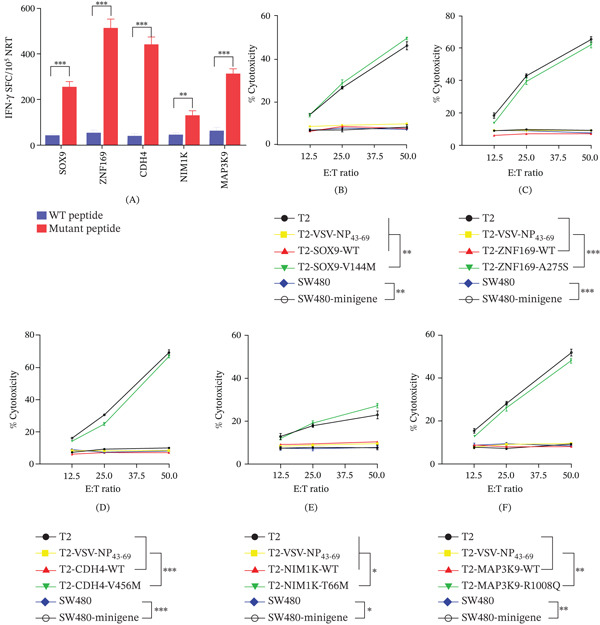
Cytotoxicity of NRTs raised by in vitro stimulation of PBLs of P1. NRTs were induced with autologous ZNF169‐A275S– and CDH4‐V456M–pulsed DCs derived from the PBLs of P1. On Day 7, after the third stimulation, the NRTs were harvested for analysis. (A) IFN‐*γ* secretion by NRT lines in response to mutated and WT peptides. IFN‐*γ*‐positive SFCs/10^5^ NRTs were detected by cytokine‐specific ELISpot. (B–F) Cytotoxicity at the indicated E:T ratios measured by a CCK‐8 kit. Peptide‐specific targets were mutated protein–pulsed T2 cells and minimally nucleated SW480 cells, whereas VSV‐NP_43-69_–pulsed T2 cells, T2 cells alone, and SW480 cells alone were used as controls. Data are expressed as mean ± SEM ( ^∗^ *p* < 0.05,  ^∗∗^ *p* < 0.01,  ^∗∗∗^ *p* < 0.001).

Although four patients were recruited for neoantigen identification, tumor tissue and blood samples collected from only P1 were sufficient for assessing the capability of NRTs to react to both mutated and WT peptides and for vaccine evaluation. Autologous DCs from P1 were used to assess the capability of NRTs to react to both mutated and WT peptides. These DCs were loaded with one of the following mutated peptides: SOX9‐V144M, ZNF169‐A275S, CDH4‐V456M, NIM1K‐T66M, or MAP3K9‐R1008Q. The purpose was to stimulate PBMCs in order to generate NRTs in vitro. NRT assays were then specifically performed for the mutated peptides in vitro. The immune responses triggered by these mutated peptides were subsequently compared with those elicited by their corresponding WT peptides. ELISpot analysis revealed that, upon stimulation with mutated peptides, NRTs released elevated quantities of IFN‐*γ*, as compared with the levels produced in response to stimulation by the analogous WT peptides (Figure 3A).

To further assess the cytotoxicity of NRTs, we employed T2 cells loaded with mutant polypeptides and SW480 cells transfected with corresponding mutant minigenes as target cells. As shown in Figure 3B–F, bulk NRTs targeting SOX9‐V144M, NIM1K‐T66M, and MAP3K9‐R1008Q—particularly those against ZNF169‐A275S and CDH4‐V456M—effectively killed target cells expressing the corresponding mutant antigens across a range of effector‐to‐target (E:T) ratios. In contrast, NRTs exhibited no significant cytotoxicity against control T2 cells (either unloaded or loaded with the irrelevant peptide VSV‐NP_43-69_) or against SW480 cells that did not express the mutant peptide. In addition, a notable reduction in TCR clonal diversity, an increase in the average frequency of clones, and a heightened TCR convergence were observed following incubation with mutated peptides. These findings suggested a significant influence of neoantigens on TCR characteristics (Figure S3A–C).

### 3.4. The Neoantigens Can Induce NRTs in the PDX Mice In Vivo

To evaluate the in vivo immunogenicity of candidate peptides, we selected ZNF169‐A275S and CDH4‐V456M from P1, whose immunogenicity had been previously confirmed in vitro, for immunization in PDX mice. On Days 0 and 7, a mixture of ZNF169‐A275S and CDH4‐V456M (100 *μ*g each) and 50 *μ*g of poly I:C was administered subcutaneously for immunization. Splenocytes were harvested 7 days after the last immunization. A portion of the splenocytes was used for IFN‐*γ* ELISpot assay, while the remaining cells were cultured for an additional 7 days with their corresponding peptides, followed by T‐cell isolation for cytotoxicity assessment.

**Figure 4:**
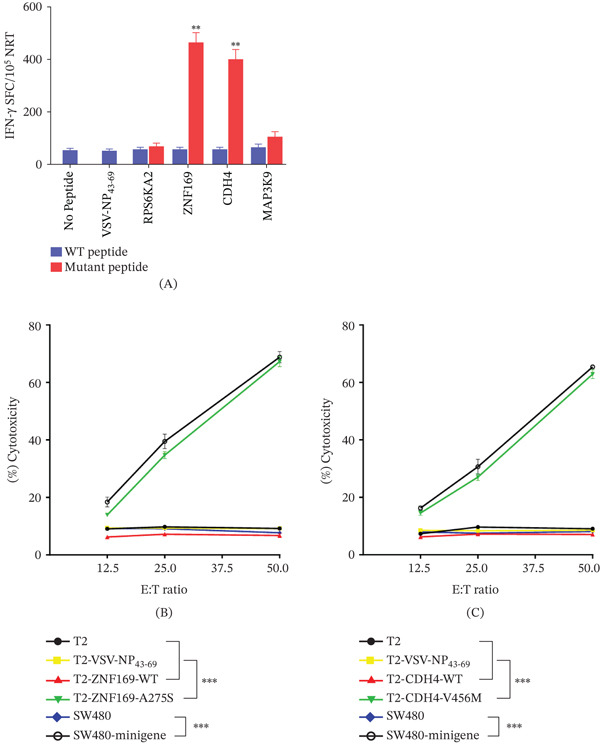
ZNF169‐A275S and CDH4‐V456M induce more efficient NRT responses than WT epitopes in PDX mice. (A) Splenocytes of mice (*n* = 5) vaccinated with mutated peptides were tested by ELISpot for the recognition of mutated peptides compared to the recognition of the corresponding WT sequences. Splenocytes from PDX mice immunized with mutated peptides were restimulated in vitro with the same mutated peptide for 7 days. (B, C) Ex vivo cytotoxicity against corresponding mutated peptide–pulsed T2 cells and minigene‐nucleofected SW480 cells was examined by CCK‐8 kit assays at the indicated E:T ratios. VSV‐NP_43-69_–pulsed T2 cells, T2 cells alone, and SW480 cells alone were used as controls. Data are presented as mean ± SEM of three independent experiments.  ^∗∗^ *p* < 0.01,  ^∗∗∗^ *p* < 0.001. Abbreviation: E:T, effector‐to‐target.

ELISpot results demonstrated that NRTs specific to ZNF169‐A275S and CDH4‐V456M from immunized PDX mice elicited a stronger IFN‐*γ* response upon stimulation with their cognate peptides compared to the WT peptide. In contrast, stimulation with medium alone, the unrelated peptide VSV‐NP_43-69_, or RPS6KA2‐P709L induced only background‐level secretion (Figure 4A). In cytotoxicity assays, NRTs—particularly those induced by ZNF169‐A275S and CDH4‐V456M—exhibited significant killing activity against T2 cells loaded with the corresponding mutant peptides and SW480‐minigene cells expressing them. No significant cytotoxicity was observed against control T2 cells (unloaded or loaded with VSV‐NP_43-69_) or SW480 cells lacking the mutant peptide (Figure 4B,C). Throughout the experiment, no adverse reactions were observed in the mice.

### 3.5. The Neoantigen Vaccines Can Induce Effective Antitumor Responses in an Autologous Humanized Mouse Model

**Figure 5:**
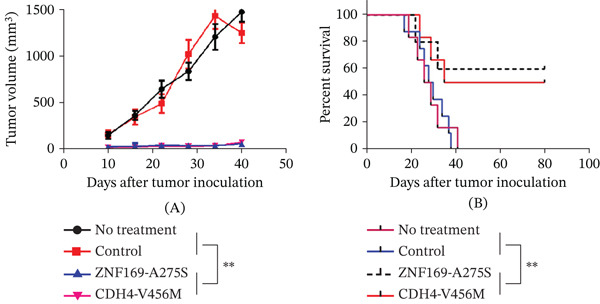
Neoantigen vaccination in the autologous humanized mouse model. The control group received only poly I:C. The “no treatment” group did not receive any treatment. (A) Tumor growth was observed every 3 days and recorded as the mean tumor volume (mm^3^). (B) Mouse survival after tumor inoculation was monitored (*n* = 10 mice per group,  ^∗∗^ *p* < 0.01).

To ascertain the potential of neoantigens as a potent vaccine for inhibiting tumor growth in vivo, two mutant polypeptides, ZNF169‐A275S and CDH4‐V456M, were selected from P1. These polypeptides had previously been identified as highly immunogenic in vitro. Autologous humanized mice were then immunized with these polypeptides. The results showed that mice vaccinated with either the ZNF169‐A275S or CDH4‐V456M polypeptide exhibited a significant delay in tumor growth (Figure 5A). Furthermore, 8/10 mice vaccinated with ZNF169‐A275S and 7/10 mice vaccinated with CDH4‐V456M demonstrated a notable long‐term survival exceeding 80 days from tumor inoculation (Figure 5B). By contrast, all mice in the control group succumbed between Days 20 and 40. Hence, these findings suggest that immunization with the ZNF169‐A275S and CDH4‐V456M peptides triggered an effective antitumor response in vivo. Tumor neoantigens are patient‐specific antigens, which are produced by unique mutations in the genes of a patient’s tumor. The personalized neoantigen can only specifically activate the patient’s own T cells to kill cancer cells. Therefore, vaccines prepared from mutation peptides produced by specific mutations in P1 should only be effective for P1 and should be ineffective for other patients. No adverse side effects were observed in the mice throughout the experiment.

## 4. Discussion

In the rapidly evolving field of immunotherapy, numerous studies have focused on the development of cancer vaccines. Neoantigens, characterized by their high immunogenicity, are not subject to thymic central tolerance [19]. Several successful Phase I clinical trials have substantially increased interest in personalized neoantigen vaccine development [16, 20–22]. Ding et al. [23] reported that a customized neoantigen peptide–loaded autologous DC vaccine was safe and effective in patients with advanced lung cancer, eliciting targeted T‐cell responses and promoting anticancer immunity. These findings suggest that personalized neoantigen vaccines represent a promising approach for treating solid tumors.

Although ICIs have improved outcomes for patients with MSI‐H–advanced CRC, those with MSS CRC, which is characterized by low mutation rates and stable MSs, do not typically respond to such therapies. Immunotherapy strategies based on neoantigens are aimed at eliminating tumor cells by enhancing host T‐cell responses. Neoantigens with high immunogenicity tend to induce stronger immune responses and have been associated with improved prognosis [24, 25], offering a potential therapeutic avenue for MSS CRC.

In this study, we proposed a workflow to predict and evaluate neoantigens in patients with MSS CRC. Neoantigens were identified for four patients, and their immunogenicity was assessed. Approximately half of the predicted neoantigens elicited T‐cell responses in patients’ PBMCs. For P1, we further compared the responsiveness of NRTs to mutated versus WT peptides and found that mutated peptides induced more efficient T‐cell responses.

Computational prediction of neoantigens remains challenging, as many predicted candidates show low immunogenicity. Traditional approaches based solely on peptide–MHC binding affinity have limited predictive accuracy. In this study, we used TruNeo, a computational tool that integrates multiple biological processes beyond binding affinity, providing a ranking of candidates with a higher likelihood of immunogenicity [12]. Consistent with prior observations, approximately half of the predicted neoantigens induced detectable T‐cell reactivity in vitro.

Although patients with MSS CRC are often considered unsuitable for immunotherapy due to low tumor mutation burden, studies have shown that they can still harbor immunogenic neoantigens [26]. A preliminary clinical study of personalized neoantigen vaccines in MSS CRC suggested that this approach is safe, feasible, and capable of inducing immune responses, potentially offering clinical benefits [10]. Our findings support the feasibility of identifying functional neoantigens in MSS CRC using whole‐exome sequencing and computational predictions.

To evaluate the in vivo efficacy of neoantigen vaccines, we used autologous humanized PDX mouse models, which are considered relevant preclinical platforms for assessing immunotherapies [17, 18, 27, 28]. By engrafting paired autologous PDX and PBMCs, we established all‐autologous humanized CRC mouse models that recapitulated tumor growth without graft‐versus‐host disease and captured interpatient variability. In these models, neoantigen vaccination significantly reduced tumor burden, supporting the therapeutic potential of this approach.

Our results demonstrate sustained neoantigen expression and robust specific immune responses in immunized mice, accompanied by significant tumor growth inhibition compared with controls. These findings suggest that personalized neoantigen‐based immunotherapy may be an effective strategy for managing MSS CRC.

### 4.1. Limitations of the Study

This study has several limitations. The sample size was small, with only four patients meeting the screening criteria for neoantigen identification. Furthermore, sufficient tumor tissue and blood samples to assess T‐cell responses to mutated and WT peptides, as well as vaccine evaluation, were available for only one patient. Due to the heterogeneity of tumors and the patient‐specific nature of neoantigens, these antigens cannot be shared across patients. Additionally, although HLA typing was performed for the patients, HLA typing of DCs was not assessed. Future studies with larger sample sizes, inclusion of DC HLA typing, and more comprehensive validation of antitumor effects in both animal models and clinical settings are warranted.

## Author Contributions

Xinqiang Yin and Jingchao Hao designed the experiment, drafted the manuscript, supervised this study, and reviewed and edited the manuscript. Wenjing Shen and Xinrong He collected and analyzed the data and revised the manuscript. Dajiang Li, Hongmei Liu, Xiao Tan, and Siyu Yang performed the ELISpot assay. All authors were involved in drafting the article or revising it critically for important intellectual content. Dajiang Li and Wenjing Shen contributed equally to this work.

## Funding

This work was supported by the Sichuan Science and Technology Program (2022JDRCO0149), the Nanchong City–North Sichuan Medical College Cooperative Scientific Research Project (22SXZRKX0014), the North Sichuan Medical College 2026 Graduate Education and Teaching Quality Project (PGJC2026034), the 2026 North Sichuan Medical College International Student Teaching Engineering Project (ISJG2026‐13), the Training Program of Innovation for Undergraduates (S202310634095, XJ202410634150, XJ202410634181, and XJ202410634158), the Basic Research Foundation of Yunnan Province Project (202401AY070001‐074), the Innovation Team Construction Project of Kunming Medical University (CXTD202203), and the Research Project on Undergraduate Educational and Teaching Reforms in Yunnan Province (JG2023001).

## Disclosure

All authors approved the final version to be published.

## Ethics Statement

This study was approved by the Ethics Committee of North Sichuan Medical College (Nanchong, China; Approval No. 1043). Informed consent was obtained from all subjects involved in the study. All animal experiments were approved by the Ethics Committee of North Sichuan Medical College (Nanchong, China; IACUC Issue No. 2022‐0038).

## Consent

Written informed consent was obtained from the patients to publish this paper.

## Conflicts of Interest

The authors declare no conflicts of interest.

## Supporting Information

Additional supporting information can be found online in the Supporting Information section.

## Supporting information


**Supporting Information 1.** Figure S1: In four CRC patients, the number of somatic mutations and corresponding predicted neoantigens in P2, P3, and P4 was examined. (A) Both whole‐exome sequencing and RNA‐seq were conducted on these patients. We identified tumor‐specific nonsynonymous somatic mutations, with the count of somatic mutations per patient indicated. (B) For each patient, neoantigens were predicted. We present the number of neoantigens, along with the classification of strong binders (*%*rank < 0.5) and weak binders (0.5 < *%*rank < 2) for each individual.

**Supporting Information 2.** Figure S2: Evaluating the immunogenicity of neoantigens from patients with CRC. Autologous PBMCs were stimulated with candidate mutated peptides every 3 days in the presence of IL‐2. On Day 10, T‐cell responses to each antigen were measured by an IFN‐*γ* ELISpot assay. The PBMCs in (A–C) were obtained from P2, P3, and P4 with CRC, respectively. No peptide (medium only) or VSV‐NP_43-69_ (STKVALNDLRAYVYQGIKSGNPSILHI) stimulation was used as a control. Data are presented as mean ± SD of three independent experiments.  ^∗∗^ *p* < 0.01 and  ^∗^ *p* < 0.05 compared with IFN‐*γ* production by PBMCs stimulated without peptide or with VSV‐NP_43-69_.

**Supporting Information 3.** Figure S3: Immune responses to personalized neoantigens in P1 with CRC. (A) TCR diversity, (B) mean clone frequency, and (C) TCR convergence were detected after treatment with the WT peptide or mutant peptide ( ^∗∗^ *p* < 0.01,  ^∗∗∗^ *p* < 0.001).

**Supporting Information 4.** Table S1: HLA alleles of four patients with CRC.

**Supporting Information 5.** Table S2: Neoantigen prediction results for four CRC patients.

## Data Availability

All data generated in the present study may be requested from the corresponding authors.
