# Supplementary figures and images for: Whole‐Exome Sequencing to Screen Personal Neoantigens With High Immunogenicity in Patients With Microsatellite Stability (MSS)–Advanced Colorectal Cancer
Source: Hum Mutat. 2026 May 4;2026:3876230. doi: 10.1155/humu/3876230 (PMC13136688; doi:10.1155/humu/3876230)

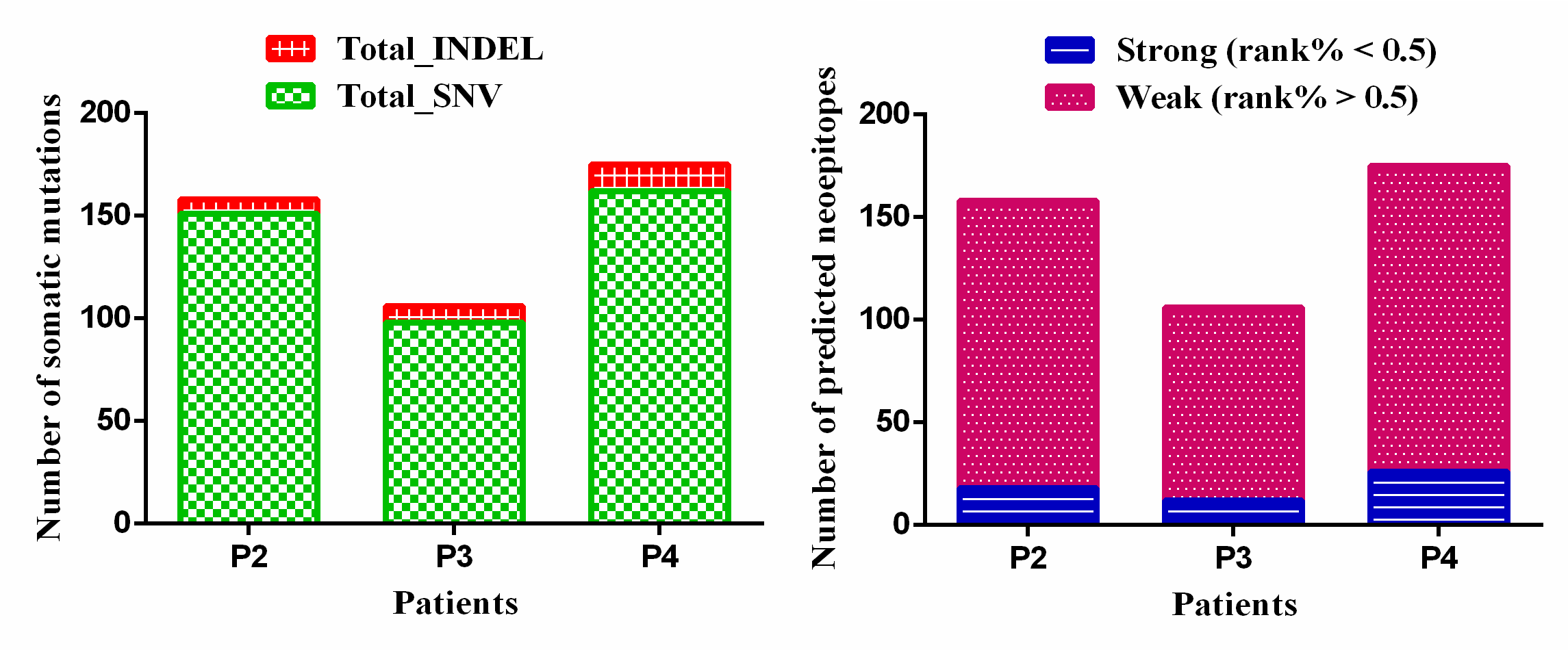

Supplement: Supplementary file 1 — Supporting Information 1. Figure S1: In four CRC patients, the number of somatic mutations and corresponding predicted neoantigens in P2, P3, and P4 was examined. (A) Both whole‐exome sequencing and RNA‐seq were conducted on these patients. We identified tumor‐specific nonsynonymous somatic mutations, with the count of somatic mutations per patient indicated. (B) For each patient, neoantigens were predicted. We present the number of neoantigens, along with the classification of strong binders (%rank < 0.5) and weak binders (0.5 < %rank < 2) for each individual. [file HUMU-2026-3876230-s001.tif]

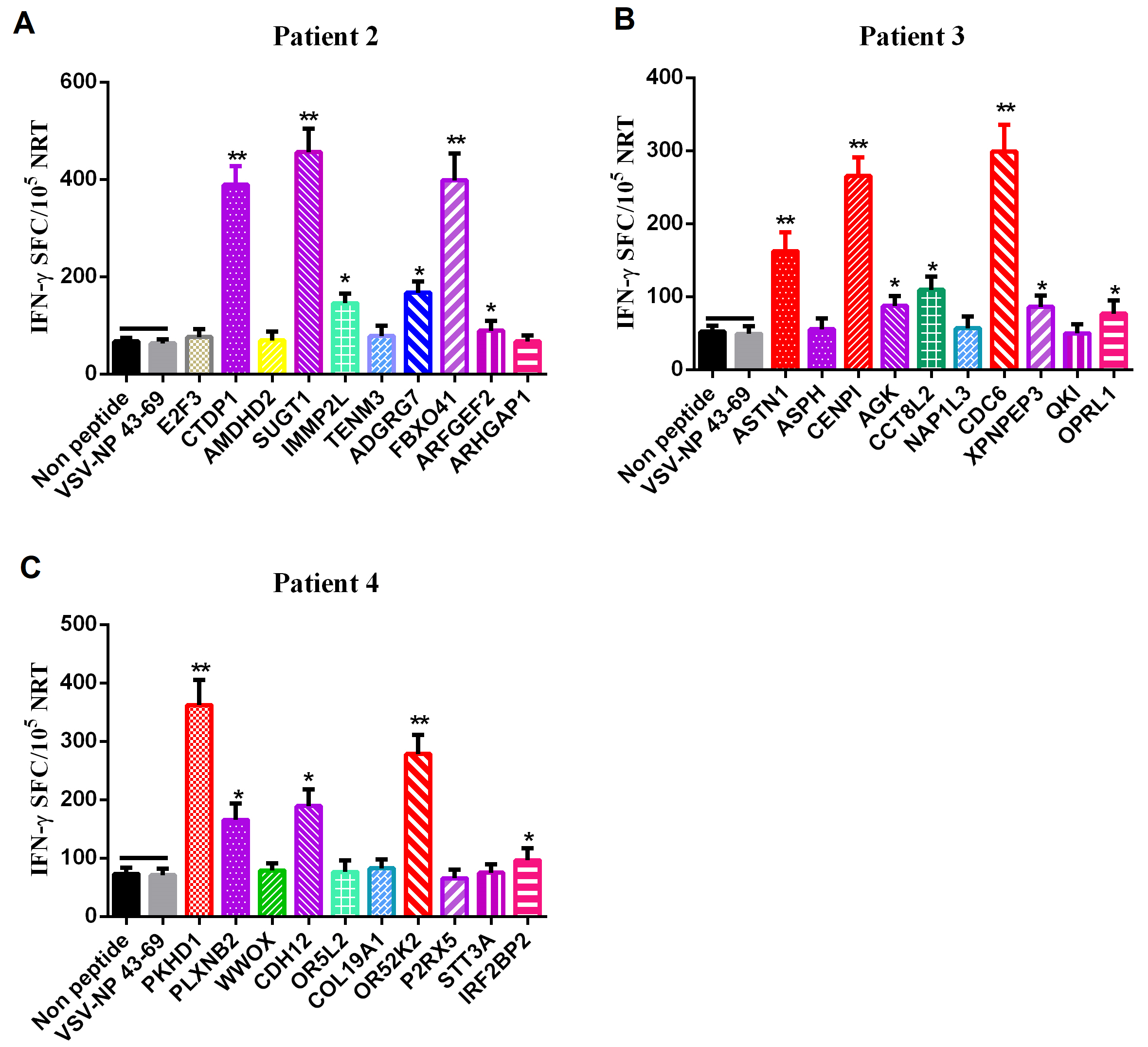

Supplement: Supplementary file 2 — Supporting Information 2. Figure S2: Evaluating the immunogenicity of neoantigens from patients with CRC. Autologous PBMCs were stimulated with candidate mutated peptides every 3 days in the presence of IL‐2. On Day 10, T‐cell responses to each antigen were measured by an IFN‐γ ELISpot assay. The PBMCs in (A–C) were obtained from P2, P3, and P4 with CRC, respectively. No peptide (medium only) or VSV‐NP43-69 (STKVALNDLRAYVYQGIKSGNPSILHI) stimulation was used as a control. Data are presented as mean ± SD of three independent experiments. ∗∗ p < 0.01 and ∗ p < 0.05 compared with IFN‐γ production by PBMCs stimulated without peptide or with VSV‐NP43-69. [file HUMU-2026-3876230-s002.tif]

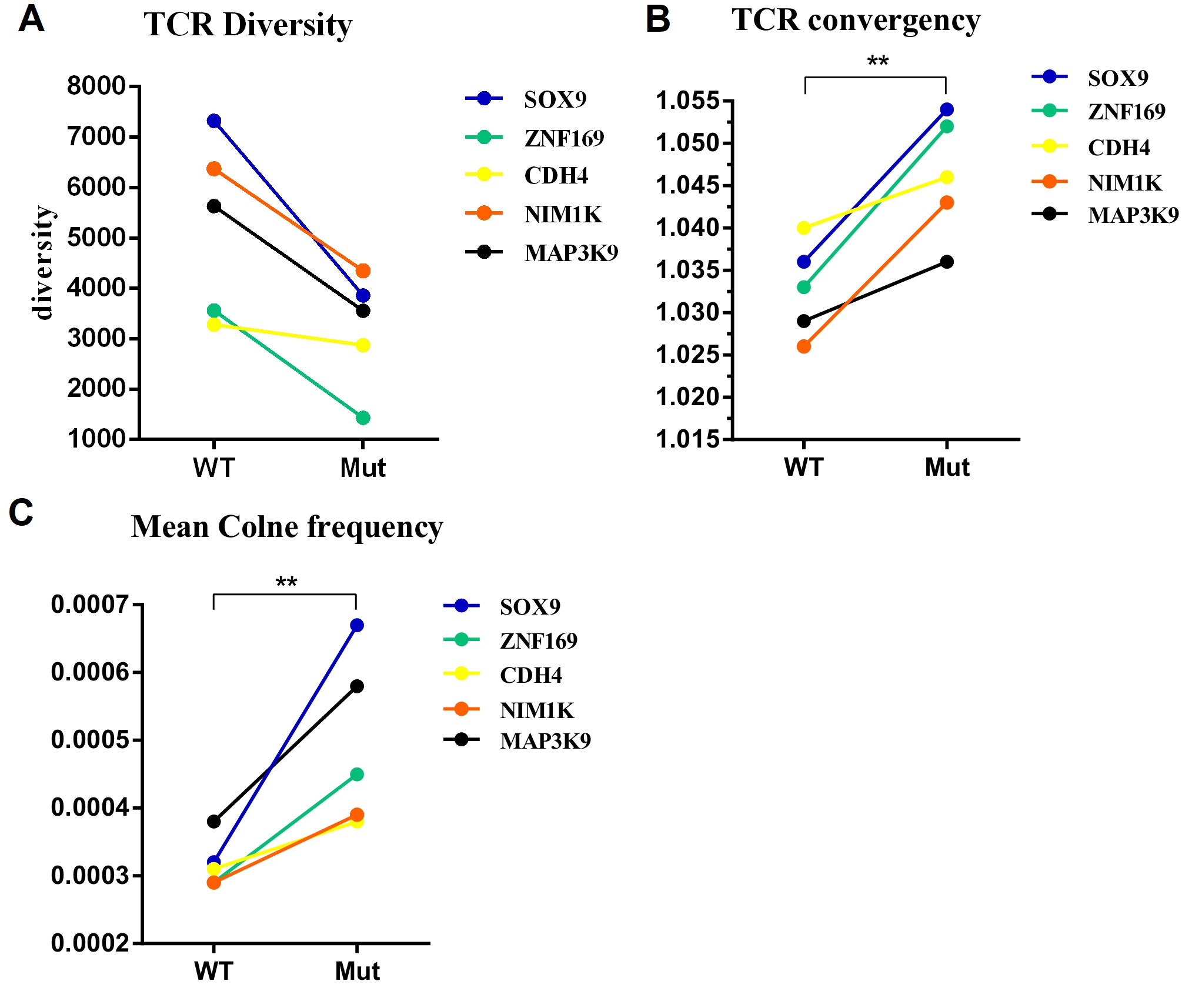

Supplement: Supplementary file 3 — Supporting Information 3. Figure S3: Immune responses to personalized neoantigens in P1 with CRC. (A) TCR diversity, (B) mean clone frequency, and (C) TCR convergence were detected after treatment with the WT peptide or mutant peptide ( ∗∗ p < 0.01, ∗∗∗ p < 0.001). [file HUMU-2026-3876230-s003.tif]
