# Supplementary material for: Whole‐Exome Sequencing to Screen Personal Neoantigens With High Immunogenicity in Patients With Microsatellite Stability (MSS)–Advanced Colorectal Cancer
Source: Hum Mutat. 2026 May 4;2026:3876230. doi: 10.1155/humu/3876230 (PMC13136688; doi:10.1155/humu/3876230)
Supplement: Supplementary file 4 — Supporting Information 4. Table S1: HLA alleles of four patients with CRC. [file HUMU-2026-3876230-s004.docx]

Supplementary table 1:

HLA alleles of 4 patients with CRC

| Patient ID | HLA I | | | HLA II | | | | | |
| --- | --- | --- | --- | --- | --- | --- | --- | --- | --- |
|  | A | B | C | DQA1 | DQB1 | DRB1 | DRA | DPA1 | DPB1 |
| P1 | A*26:01 A*33:03 | B*13:01 B*44:02 | C*03:04 C*05:01 | DQA1*05:01 DQA1*03:01 | DQB1*03:01 DQB1*03:01 | DRB1*11:01 DRB1*11:01 | DRA*01:01 DRA*01:01 | DPA1*01:03 DPA1*01:03 | DPB1*21:01 DPB1*05:01 |
| P2 | A*26:01 A*11:01 | B*40:01 B*51:01 | C*07:02 C*14:02 | DQA1*03:01 DQA1*05:01 | DQB1*03:03 DQB1*02:02 | DRB1*09:01 DRB1*11:01 | DRA*01:01 DRA*01:01 | DPA1*02:02 DPA1*02:02 | DPB1*105:01 DPB1*05:01 |
| P3 | A*02:07 A*33:03 | B*15:01 B*58:01 | C*03:04 C*05:04 | DQA1*03:01 DQA1*01:02 | DQB1*06:02 DQB1*06:02 | DRB1*15:01 DRB1*16:08 | DRA*01:01 DRA*01:01 | DPA1*01:03 DPA1*01:03 | DPB1*02:01 DPB1*02:01 |
| P4 | A*33:03 A*23:01 | B*49:01 B*35:01 | C*03:03 C*07:01 | DQA1*03:01 DQA1*05:01 | DQB1*03:02 DQB1*02:01 | DRB1*13:01 DRB1*11:01 | DRA*01:01 DRA*01:01 | DPA1*01:03 DPA1*01:03 | DPB1*03:03 DPB1*02:02 |
